# Spindle orientation and epidermal morphogenesis

**DOI:** 10.1098/rstb.2013.0016

**Published:** 2013-11-05

**Authors:** Anita Kulukian, Elaine Fuchs

**Affiliations:** Laboratory of Mammalian Cell Biology and Development, Howard Hughes Medical Institute, The Rockefeller University, New York, NY 10065, USA

**Keywords:** asymmetric cell division, symmetric cell division, spindle orientation, cell fate

## Abstract

Asymmetric cell divisions (ACDs) result in two unequal daughter cells and are a hallmark of stem cells. ACDs can be achieved either by asymmetric partitioning of proteins and organelles or by asymmetric cell fate acquisition due to the microenvironment in which the daughters are placed. Increasing evidence suggests that in the mammalian epidermis, both of these processes occur. During embryonic epidermal development, changes occur in the orientation of the mitotic spindle in relation to the underlying basement membrane. These changes are guided by conserved molecular machinery that is operative in lower eukaryotes and dictates asymmetric partitioning of proteins during cell divisions. That said, the shift in spindle alignment also determines whether a division will be parallel or perpendicular to the basement membrane, and this in turn provides a differential microenvironment for the resulting daughter cells. Here, we review how oriented divisions of progenitors contribute to the development and stratification of the epidermis.

## 1. Introduction

**Figure 1.**
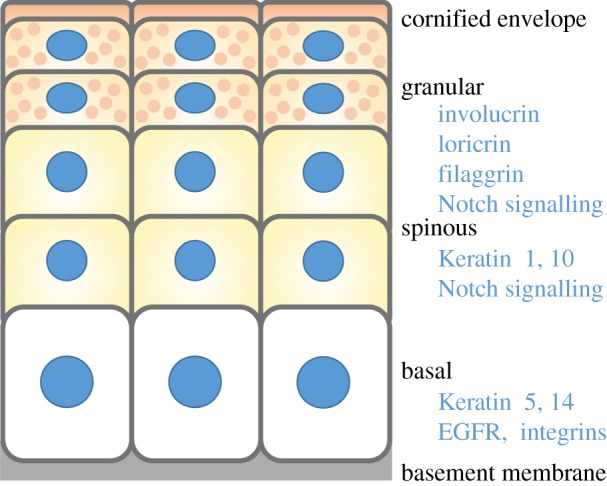
Epidermal organization. The stratified epidermis consists of basal, spinous and granular layers, and the cornified envelope, assembled upon the basement membrane. Each layer expresses distinct keratin filaments and is receptive to different signalling pathways.

The interfollicular epidermis of adult mammalian skin is a stratified epithelium, the outermost layer of which is the body surface (figure 1). Its function is to act as a barrier to keep harmful microbes out and retain essential fluids. Throughout life, the epidermis must constantly rejuvenate and replace dying or worn cells with fresh ones. It does so through a process referred to as homeostasis, in which cells from a single inner proliferative (basal) layer periodically withdraw from the cell cycle, commit to differentiate and move upward. In the first stage of differentiation, spinous cells remain transcriptionally active; in the absence of cycling, these enlarged cells produce an extensive mechanical infrastructure, composed of copious amounts of keratin filaments linked in a network to robust desmosomal intercellular junctions. As cells enter the next stage, the granular layer, they produce lamellar granules packed with lipid bilayers, and they deposit an elaborate array of proteins, the cornified envelope, just beneath the plasma membrane. In the last stage of terminal differentiation, all metabolic activity ceases, and calcium influx triggers transglutaminase enzymes to cross-link the envelope proteins with indestructible γ-glutamyl-*ɛ*-lysine bonds. These cells lose their organelles including the nucleus, extrude the lipids on the cornified envelope scaffold and effectively seal the skin surface. Eventually, these dead squames are sloughed from the skin surface, and replaced by new cells differentiating upward.

In the adult epidermis, tissue balance is maintained when the rate of cells departing the innermost layer and committing to terminally differentiate is off-set by cells reaching the skin surface and being sloughed. However, during embryogenesis, the skin begins as a single layer of unspecified progenitors, which must proliferate rapidly to keep up with the growing embryo, but at the same time stratify to produce the mature epidermis. The single layer must also generate the epidermal appendages de novo, most notably, the hair follicles; this necessitates an even larger output of cells during the process.

The proliferative capacity of the epidermis is maintained by cells’ ability to attach to an underlying basement membrane, rich in growth factors and extracellular matrix (ECM) ligands. The basement membrane serves as a platform for basal cells to adhere. It does so through integrins α_3_β_1_ and α_6_β_4_, both of which bind to laminin V, the cornerstone of the basement membrane that separates epidermis from underlying dermis. Basal progenitors adhere to their neighbours through E-cadherin-mediated adherens junctions. Even when the embryonic epidermis exists as just a single layer, the cell–substratum and cell–cell junctions give the basal layer its unique apico-basal polarity and proximity to growth-promoting factors that distinguish it from its suprabasal terminally differentiating progeny.

**Figure 2.**
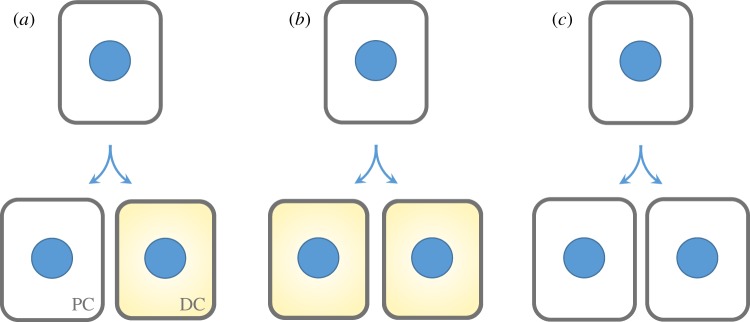
Balancing stem cell divisions with cell fate. (*a*) Progenitors in the basal layer of the epidermis can divide asymmetrically to yield two cells that are differentially fated to become a progenitor cell (PC) and a differentiating cell (DC). Alternatively, they can divide symmetrically to produce two cells that both either differentiate, as in (*b*) or maintain their progenitor fate, as in (*c*). Asymmetric divisions would maintain tissue balance, as in homeostasis, whereas symmetric divisions would alter the stem cell population within the tissue, as in processes such as ageing, wound healing or hyperproliferative disorders, including cancer.

Whether during morphogenesis or in the adult, tissue maintenance in the epidermis is the responsibility of stem cells, as it is in most if not all tissues of the body. Stem cells ultimately must divide in such a way as to self-renew while also giving rise to a population of cells with more specified function. Mechanistically, there are three possible ways in which stem cells could divide to influence tissue balance (figure 2). If stem cells asymmetrically divide to generate one progenitor and one terminally differentiating daughter, they would always maintain exact tissue balance. If they generate two terminally differentiating daughters, the pool of stem cells would be in a state of decline; if they generate two proliferative daughters, the pool of stem cells would be expanding. If stem cells have the capacity to undergo any one of these division types, then their flexibility would be maximal, enabling them to tailor their choice to suit the particular needs of each tissue and situation.

In normal homeostasis where tissue balance is maintained, stem cells must either always divide to generate a stem cell and a terminally differentiating daughter, or balance precisely the two types of symmetric divisions. Although stem cells are typically viewed to divide asymmetrically, there are two mechanistically distinct ways by which this can be achieved. In a classical sense, a true asymmetric cell division (ACD) unequally partitions macromolecules, organelles and even cell size. However, in the field of stem cell biology, the term ACD is often more broadly interpreted to include any type of division that yields a differential fate outcome. This can happen, for instance, not only by asymmetric partitioning of proteins during the act of division, but also by a symmetric division in which one daughter is either displaced from the stem cell niche and/or otherwise exposed to external differentiation signals relative to the other daughter.

Despite much speculation as to the implementation of ACDs in adult stem cell biology, much of what is known about ACDs comes from studies of embryogenesis. The epidermis is no exception, where embryonic analyses have shown that epidermal ACDs are accomplished by orienting the plane of division relative to the basement membrane to yield two cells within different strata with differing response to Notch signalling. Here, we review what is known about ACDs in the developing epidermis, and extrapolate this information to what might be anticipated in adult stem cell niches.

## 2. Stratification in the epidermis: balancing proliferation and differentiation

Epidermal identity is specified by embryonic day (E) 9.5, when it transits from a single layer of surface ectoderm to a tissue expressing Keratin 5 and 14. Cell divisions occur within this lateral plane, allowing the tissue to expand to meet the rapid growth of the embryo. Starting at E12.5, the epidermis ceases its exclusive lateral expansion and begins to also stratify into suprabasal layers committed to terminal differentiation.

**Figure 3.**
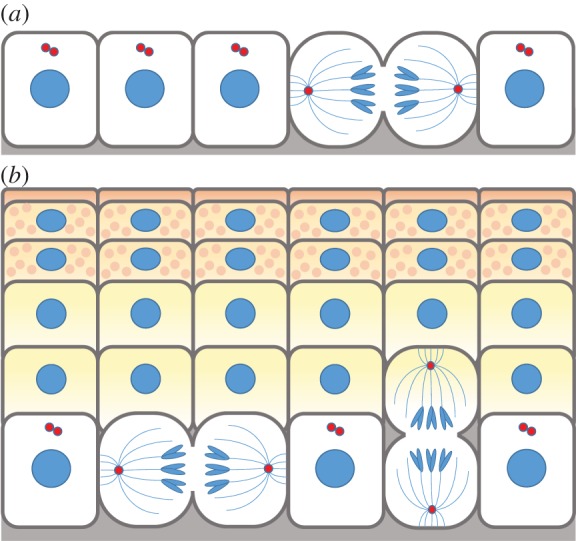
Stratification of epidermis through oriented divisions. (*a*) At early stages of embryonic development, progenitor cells of the epidermis divide with their spindle oriented parallel to the basement membrane. (*b*) As stratification and differentiation progresses, perpendicular oriented divisions begin to dominate, representing up to 70% of all divisions through these critical stages of embryogenesis. Perpendicular divisions generate a basal progenitor cell and a differentiating cell positioned in the suprabasal layer. These differential cell fate acquisitions define the ACDs of the epidermis.

Morphological studies gave the first clues that as early basal epidermal progenitors begin to develop tissue, they reorient their mitotic spindles; by E15.5, greater than 70% of mitotic spindles orient perpendicular rather than parallel relative to the underlying basement membrane (figure 3) [1–4]. This preferred asymmetric orientation of spindles is maintained until birth when the epidermis is fully mature. Postnatally, perpendicular spindle orientation has not been observed in the backskin of adult mice [5]. However, as hair follicles form and mature, the epidermis thins and proliferation wanes, and thereafter the hair coat plays the lion's share of protection to the body surface.

Only a few divisions occur suprabasally, and these divisions are restricted to the first suprabasal layer and in a brief window of early embryonic epidermal stratification, coincident with the timing at which a paucity of suprabasal cells exists. Both daughters of such divisions appear to be slated for differentiation, as judged by the expression Keratin 1, a differentiation marker [2]. This has led to the hypothesis that these atypical suprabasal symmetric divisions probably function to fulfil the peak demands of the rapidly expanding embryo. That said, the limited numbers of suprabasal divisions are unlikely to account for the bulk of stratification and differentiation that must occur in these early stages of development. This leaves the innermost, basal epidermal layer both to fuel the generation of sufficient new proliferative progenitors to keep up with embryo expansion, as well as to generate the necessary additional stratifying cells that will progress to terminally differentiate.

*A priori*, basal progenitors could fuel the production of basal and suprabasal cells by one of several modes of cell division. In consideration of the first possibility, a basal cell can divide laterally. If it divides symmetrically to generate two basal cells, as in figure 2, then local heterogeneity in the surrounding microenvironment would then have to drive intracellular changes that would lead to the eventual delamination of some of these daughter cells. If on the other hand the basal cell divided asymmetrically, thereby partitioning its proteins differentially, one basal cell would need to inherit more progenitor factors, e.g. integrins and growth factor receptors, while the other would need to receive a preferential dose of differentiating factors, thereby resulting in its delamination and differentiation.

The asymmetry achieved by a perpendicular spindle orientation, as seen in the other 70% of embryonic basal cells, is maintained even after cleavage furrow formation. Thus, although video imaging will be necessary to establish whether these divisions actually displace one of the two daughters, confocal microscopy coupled with mitotic staging favours such a model [4]. Additional support comes from lineage tracing experiments with K14-CreER×Rosa–lox–Stop–lox-GFP mice. In this experiment, tamoxifen is topically applied to transiently induce Cre recombinase in a small number of (K14+) basal epidermal cells. These cells then excise the stop codon, resulting in permanent expression of GFP in the cells and all their subsequent offspring. Such clonal tracings demonstrate that the outcome of a perpendicular division is a K10-negative basal cell and a K10-positive (and thus differentiating) suprabasal cell [3]. While it is simpler to conceptualize how asymmetric fates are achieved in two daughter cells in perpendicular rather than parallel divisions, whether the division symmetrically or asymmetrically partitions its proteins cannot automatically be determined by spindle orientation. If the division process displaces one of the two basal daughters into the suprabasal compartment, microenvironmental cues could still be the driving force underlying asymmetric fate acquisition. That said, asymmetric partitioning of proteins in a division associated with perpendicular spindle orientation becomes particularly attractive, since the basal cell will be automatically primed to preferentially inherit the bulk of the basal membrane surface proteins and all of their associates. This would include integrins and growth factor receptors, thereby fuelling a progenitor status.

What seems to distinguish the epidermis is that its cellular divisions are not confined to a single mode or a single orientation. Both short-term pulse labelling and long-term lineage tracing experiments in embryonic and adult mouse tail epidermal progenitors have shifted the paradigm of how progenitors divide in the epidermis. By correlating the size of each labelled clone and the presence of basal and suprabasal cells within those clones, it was demonstrated that basal cells can divide either symmetrically to yield two basal cells or asymmetrically to produce a basal cell and a suprabasal cell. Thus, epidermal progenitors are not committed to dividing either symmetrically or asymmetrically, but the choice in division is somewhat stochastic in nature [3,5,6], most likely tailored to suit the shifting needs of the dynamic skin epithelium. Therefore, understanding how a single cell type can choose between an asymmetric and symmetric division can elucidate the molecular mechanisms that orient divisions within a particular tissue and help identify the cues which prompt a particular orientation during division.

## 3. Regulation of spindle orientation and asymmetric cell division

Much of what we know about the molecular underpinnings of ACDs and spindle orientation comes from *Drosophila* and *Caenorhabditis elegans*, where studies in the development of neural progenitors, germline stem cells and zygotes have all been shown to undergo divisions that asymmetrically distribute proteins and organelles into the daughter cells. While oriented divisions have been observed in a variety of organisms and their diverse tissues [7], the expansion of these studies to lumen-forming cyst cultures and the morphogenesis of the mouse brain and skin in particular have demonstrated that despite varying degrees of homology between invertebrate homologues and their mammalian counterparts, these components serve similar functions in mediating ACDs, though with mechanistic differences.

As a universal but somewhat generalized theme built upon the *Drosophila* neuroblast model for closest comparison, the cell cortex is polarized by the asymmetric distribution of the Par complex consisting of Bazooka/Par3–Par6–atypical protein kinase C (aPKC) at the cell periphery, along with the Gα*_i_* subunit of heterotrimeric G proteins [8–13] . During mitosis, two key proteins are recruited to these polarized cortical sites: Inscuteable (mInsc in mammals) and Pins (LGN in mammals) [8,11,14–16]. Insc/mInsc and Pins/LGN orient the mitotic spindle through the cortical capture of astral microtubules via the microtubule binding protein Mud/NuMA and through the microtubule pulling forces of its interacting partner, the motor complex Dynein/Dynactin [17–23]. The cleavage plane then influences the identity and fate the two daughter cells will adopt, because it is coupled with the asymmetric distribution of cell fate determinants.

**Figure 4.**
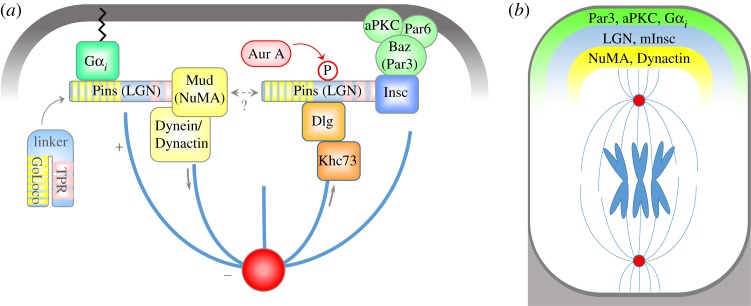
Molecular mechanism orienting the mitotic spindle. (*a*). In *Drosophila* neuroblasts, Pins interacts with cortical Gα*_i_* and binds to Mud. Mud and Dynein orient the spindle via astral microtubules. LGN also binds to Insc, in a complex with Par3. A second mechanism to orient the spindle has been discovered, in which Aurora A phosphorylates the linker region of Pins to recruit Dlg and Khc73. (*b*) In epidermal basal cells undergoing ACD, LGN, mInsc, NuMA and Dynactin are recruited to the apical cortex in mitosis, and function in orienting the spindle perpendicular to the basement membrane.

By teasing apart the biochemical interactions within the ACD machinery, progress has been made in understanding how the spindle is anchored in line with cortical polarity cues. Much of it is dependent on the multi-domain structure of Pins/LGN (figure 4). In the absence of binding partners, Pins/LGN's amino-terminal TPR repeats interact with the carboxy-terminal's GoLoco domains, imparting a closed conformation [17,24]. The binding of either Mud/NuMA to the TPR domain or of Gα*_i_* to the GoLoco domains alters the conformation of Pins/LGN, allowing it to form a tripartite complex with both proteins [17,24]. Pins/LGN is also recruited to the apical surface through a different set of interactions involving Par3. Par3 can directly bind to Insc [8,10], which also interacts with Pins/LGN via the TPR domain [15,25]. While it was initially believed that Insc could be incorporated into this complex, recent findings have shown that binding of Pins/LGN to Insc is mutually exclusive to its interaction with Mud/NuMA [25]. That said, this does not rule out a potential role for Insc in participating in the anchorage of the spindle, as this cortical astral microtubule pad is no doubt a multimeric protein complex, composed of many subunits of each type. Moreover, there could be additional as yet unidentified proteins that facilitate connections between the Gα*_i_*–LGN and Par3–Par6–aPKC–Insc complexes. An important question in the field is to what extent each of these complexes act independently versus coordinately in governing spindle orientation and ACDs.

An additional mechanism orienting the spindle has been identified through the induced polarization of *Drosophila* S2 cells. There, Aurora A-dependent phosphorylation of the internal linker domain within Pins/LGN enhances its TPR domain interactions [26]. This precipitates the recruitment of the tumour suppressor Dlg, and connects astral microtubules through the kinesin Khc73 (Kif13b in mammals) [26–29]. Dlg was later found in complex with Insc–LGN, and excluded from the LGN–NuMA interaction [30]. These sets of interactions, on the surface, seem to bisect cortical anchorage into two spindle orientation pathways, one which includes Par3–Insc–LGN (and potentially Dlg), and another with Gα*_i_*–LGN–NuMA. However, there is ample evidence demonstrating that the two impinge upon the other, but the molecular details are currently absent from our understanding.

Which of these pathways are used in orienting the spindle apico-basally in epidermis? The Par3 (Par3–Par6–aPKC) complex localizes to the apical domain which is directly opposed to the basement membrane [2]. Gα*_i_*3, mInsc, LGN, NuMA and even the p150Glued/Dctn1 subunit of the Dynein/Dynactin complex are all enriched at the apical cortex in asymmetrically dividing cells [2–4]. Depletion of LGN, NuMA and Dctn1 by lentiviral-mediated delivery of shRNAs to the E9.5 embryo surface epithelium reduces the number of perpendicular asymmetric divisions in developing epidermis and results in a consequent thinning of the suprabasal layers and impaired barrier function by birth. These findings establish a functional role for these proteins during epidermal stratification and differentiation. Reduction of these components also demonstrates that spindle orientation in the epidermis is established cell autonomously—shRNA-uninfected cells display a normal distribution of ACD to symmetric divisions [4]. However, homology does not automatically dictate a role in ACDs of the skin, as AGS3, the other mammalian LGN isoform which is required for ACDs in neural progenitors [31], does not seem to be required for asymmetric divisions in epidermis—it does not polarize to the apical membrane and its depletion does not result in differentiation defects [4]. Localization assays have also confirmed that a hierarchy of interactions of Gα_*i*_ > LGN > NuMA > Dctn1 is necessary to orient the spindle perpendicularly in the epidermis.

While a clear role has been demonstrated for LGN and NuMA in asymmetric divisions, it is less clear as to what precise function mInsc plays in orienting the spindle in the epidermis. By binding to Par3, it acts as a direct link to cortical polarity. Both mInsc and Par3 are required for the ACDs during mammalian neocortex development [32,33]. The Par3 conditional knock-out mice develop a relatively normal epidermis [34], while direct examination of how the depletion of mInsc can affect the development of the epidermis remains unknown. By contrast, elevated levels of mInsc, accomplished either by inducible expression of a transgene or by lentiviral introduction in a mouse, promotes up to a 20% increase in observed ACDs in embryonic tissue when LGN is also present [3,4]. Surprisingly, this increase is only temporary, and what follows is a reduction in ACDs after 3 days of overexpression. Thus, prolonged mInsc expression is not sufficient to maintain the increase in ACD. Inspection of asymmetrically dividing mitotic cells pointed to an uncoupling of mInsc crescents from cortical NuMA, revealing a requirement for NuMA to align with mInsc at the cell cortex to position the spindle apico-basally [3]. However, since NuMA does not interact with mInsc directly, and the binding of LGN with mInsc is mutually exclusive from its interaction with NuMA as described above, how the Gα*_i_*–LGN–NuMA pathway interacts with cortical Par3–mInsc–LGN to orient the spindle still remains a mystery, underscoring the importance of resolving this important question in the future.

It is currently also unknown whether a Dlg-dependent spindle orientation mechanism functions in the epidermis. However, Dlg is conserved in mammals, where its four isoforms Dlg1–4 and a more distant relative (Dlg5) belong to a group of membrane associated guanylate kinase proteins which have been implicated in epithelial polarity, junctional formation and planar cell polarity [35]. Interestingly, Dlg1 translocates to the cell cortex upon high calcium-mediated differentiation induction in cultured keratinocytes [36]. The relative contribution of Dlg1 in epidermal ACDs can now be explored, since the reporting of a Dlg1-null mouse model [37]. More interesting is Dlg5, which was recently shown in lung development to function in the apical maintenance of aPKC [38].

These data suggest that Dlg5 (and possibly other Dlgs) may function in regulating apical polarity complexes in mammalian epithelial tissues. However, since NuMA depletion in epidermis decouples the spindle pole from cortical cues with almost complete penetrance [4], it seems unlikely that there is another dominant and fully redundant pathway reliant upon Dlgs that would link the astral microtubules/spindle poles to the cortex. Also arguing against the *Drosophila* Dlg pathway is the timing of its activation: the Pins^Linker^–Dlg–Khc73 pathway is already activated in prophase by the activity of Aurora A, whereas the epidermal spindle is not oriented until prometaphase (more on this below).

## 4. Apico-basal polarity, spindle orientation and the balance of epidermal growth and differentiation

Several lines of genetic evidence suggest that apico-basal polarity plays a key role in establishing the location of Par3/aPKC and the apical crescent of LGN and mInsc. Conditional genetic ablation of β_1_ integrin, which is required for assembly of the basement membrane [39], randomizes cortical LGN–NuMA localization. Loss of the adherens junction protein α-catenin in the epidermis compromises cell–cell adhesion, and in this scenario, the cortical crescents of LGN and NuMA no longer form at all. The consequence of both deficiencies is randomization of the spindle orientation and a severe disruption in the balance between progenitors and differentiating cells within the developing epidermis. By contrast, the loss of β_4_ integrin (a component of hemidesmosomes) or desmoplakin (a component of desmosomes) has no comparable effect on LGN and mInsc localization [2].

Since β_1_ integrin and α-catenin-mediated junctions associate preferentially with actin networks, while hemidesmosomes and desmosomes prefer intermediate filament networks, these results suggest a special importance of the cortical actin network and the three-dimensional architecture of the epidermis for proper spindle positioning. Indeed, when the cortical actin network is perturbed, defects in spindle orientation are among the earliest consequences. This was first discovered by loss-of-function mutations in *SRF*, a transcriptional regulator sensitive to G-actin levels and whose target genes encode a number of actin binding proteins, as well as actin itself. Without SRF, cortical actin is diminished and localization of LGN and NuMA as well as spindle polarization are all compromised [40].

Part of these defects is due to the inability for proper cell shape changes to occur during mitosis. Moreover, one of the consequences of the loss of cortical actin in SRF-conditionally null epidermis is that the location and phosphorylation of ERM proteins (Ezrin/Radixin/Moesin) is compromised [40]. Following this defect, Par3 localization is altered, and so is interphase centrosome positioning. Recent studies in Caco2 cells have demonstrated that throughout much of the cell cycle, centrosomes remain tethered underneath a cortical Ezrin ‘cap’ which is apically restricted there by the tumour suppressor Merlin [41]. Loss of cortical localization of Ezrin results in the loss of cortical tethering of centrosomes, misoriented spindles during ACDs and failure to form a proper lumen. It is possible that the same pathway for centrosomal positioning is also at play in the epidermis because Ezrin is also localized to the apical surface of basal cells [41]. Dividing basal cells which are null for Merlin have randomized spindle orientation, resulting in a hyperproliferative basal layer and differentiation defects [42]. It remains to be seen whether defects in spindle orientation can be ascribed to centrosome positioning or to the direct alteration of apico-basal polarity, but both are likely to have complementary roles.

## 5. Asymmetric versus symmetric divisions: wherein lies the choice?

While much headway has been made in understanding how the spindle is locked into position during asymmetric divisions, we still have only a few minor hints of how spindles in lateral divisions become oriented and how the choice to switch to a perpendicular spindle orientation is made. In two-dimensional analyses, depletion of NuMA, LGN and Dctn1 all create a bias towards lateral spindle orientation [4], suggesting that planar spindle orientation might be a default pathway albeit with its own positional cues. The expression and stability of ACD machinery itself is also insufficient, since NuMA is present at both spindle poles of most dividing cells, yet not at the cortex of cells undergoing lateral divisions. It is, however, enriched at the apical pole during a perpendicularly oriented division [2]. We do not fully understand the nuances of NuMA and LGN localization, but all signs point to them as crucial in unravelling the molecular mechanism guiding divisional orientation.

Another possibility might be inherent to the positioning of the centrosomes themselves. Centrosomes are the underlying organelles of each spindle pole and function as microtubule organizing centres. Duplicated centrosomes are often localized within close proximity to each other, and their separation towards opposite sides of a cell is required for the formation of a functional bipolar spindle in mitosis in vertebrates. Separation can be accomplished either by the migration of one pole in relation to the other, or by the simultaneous separation which is then followed by rotation of the spindle into position. As observed during the migratory positioning of centrosomes in *Drosophila* male germ stem cells (GSCs) [43] and neuroblasts [44], positioning of the spindle poles occurs prior to mitosis, with one centrosome remaining anchored against the cell cortex, whereas the other centrosome has greater mobility throughout the cell. In these systems, spindle orientation is established by the location of the migratory centrosome [44–46]. If this occurred in the epidermis, it would obligatorily force the spindle into a perpendicular orientation, because centrosomes of basal cells are apically positioned throughout interphase. However, this is not the case. As shown by immunostaining and by tracking fluorescently labelled centrosomes, both centrosomes of basal cells remain apically positioned during the early stages of mitosis, even as the mitotic spindle is forming. It is in prometaphase that separation of the poles is initiated [3,4,47], and in due time the spindle is rotated into position, either into a planar or apico-basal orientation. Because centrosomal positioning is not pre-determined prior to mitotic entry, it may be one of the underlying reasons why basal keratinocytes have the flexibility to align their spindle either laterally or perpendicularly. Unravelling the players that aid in centrosomal anchoring may prove important in understanding how the spindle poles then reposition themselves in alignment with cortical cues and in sync with the cellular decision of how to divide.

## 6. Inheritance of maternal centrosomes

Beyond simply positioning the spindle, it is tempting to speculate that centrosomes might have additional functional roles in regulating ACDs. Centrosomes, like DNA, undergo cell cycle-dependent replication and segregation, with each new daughter cell inheriting a single copy. Each duplicated centriole undergoes a phase of maturation in the following cell cycle, during which time it obtains distal and subdistal appendages and accumulates pericentriolar material (PCM). Thus, the two centrosomes of a cell contain differently aged mother and daughter centrioles, which can be distinguished by protein content and function.

Asymmetric inheritance of aged centrosomes has been observed in *Drosophila* male GSCs, in which the mother centrosome is invariably inherited [48], and in neuroblasts, where it is the immature daughter centrosome that is inherited [49,50]. Inheritance of a specific centrosome has not yet been explored in the epidermis, but it has been observed in the developing neocortex of the mouse. There, depletion of Ninein, a subdistal appendage protein enriched on, and a marker for, the mother centriole [51], resulted in the depletion of progenitor neurons, with a concomitant increase in the number of differentiated ones [52].

It is unclear why inheritance of a particular centriole promotes stemness in these cells. It is possible that it could be due to unique proteins or RNAs that localize to the appendages or the PCM [53] that may regulate cell fate, including Ninein itself, and that are disproportionally shuttled via centrosomes to progeny cells, providing a novel mechanism by which to segregate cell fate determinants. It is a tempting speculation, since in surf clam, unique centrosomal RNAs, including those for morphogens, are segregated into cells with a clear cell fate path [54]. However, the overlooked fact is that centrosome maturation has a functional consequence: the appendage-bearing older mother centrosome is better able to anchor to the cortex and promote more robust nucleation of microtubules, with greater stabilization of astral microtubules [55].

Ninein itself is thought to promote a more robust microtubule lattice—and anchor the centrosome at the minus end [56,57]. In the epidermis, Ninein is associated with the centrosomes in basal cells, where it may promote microtubule nucleation as well. However, Ninein, along with centrosomal components Lis1, Clip170 and Ndel1, relocalizes from centrosomes to the desmosomes in stratified layers, where it stabilizes desmosome-associated acentrosomal cortical microtubule network [47,58]. Relocalization of Ninein has also been observed during differentiation of mouse neurons and in cochlear epithelial cells [59,60]. This yields the possibility that differentiation defects arise not from inheritance errors *per se*, but from either microtubule anchoring defects or desmosomal dysfunction, or both, rather than the inheritance of a specific cell fate determinant. Additional research will help resolve this outstanding question.

In addition to spindle anchoring and microtubule nucleation, centrosomes also form the basal body from which the primary cilium is extended. The cilium serves as a cellular antenna to transduce signalling events. The appendages of the mother centriole also aid in the docking of the centrosome to the cell periphery, and it is the mother centriole from which the primary cilium is extended [61]. Since each daughter cell inherits either a mother or a daughter centriole, the inheritance of a differently aged centriole can create a differential (and temporally regulated) response to signalling molecules. Indeed, a role for cilia in Notch signalling has been described in the epidermis [62].

## 7. Notch as an effector for cell fate determination

For most well-characterized invertebrates systems undergoing ACDs, cell fate acquisition is accomplished by an accompanying asymmetric inheritance of cell fate determinants. In neuroblasts, these are exemplified by Prospero, Brat and Numb, which function in transcriptional activation, transcriptional inhibition and Notch signalling antagonism, respectively. They localize to the basal cortex in mitosis, and the angle of division determines which daughter will inherit these factors to promote differentiation [63–65].

In the epidermis, the extent to which cell fate acquisition is linked to cell divisions is less clear. In the embryonic epidermis, ACDs appear to be coupled to Notch signalling [4], but the underlying mechanisms are unknown. Asymmetric localization of the Notch inhibitor Numb has only been reported in laterally dividing cells of adult epidermis, and even there, a functional role for Numb or even its asymmetric partitioning to daughter epidermal cells remains to be demonstrated [4,5]. Prospero and Brat also have vertebrate homologues, but whether they are asymmetrically inherited or have a role in epidermal development has not yet been explored. In order to localize to the basal cortex, these determinants must bind to the adaptor protein Miranda [66–69], and no vertebrate homologue of Miranda has been identified. In the absence of Miranda or polarized distribution of determinants, it remains to be seen whether the mechanism of partitioning cell fate determinants is conserved or even necessary.

There are ample examples in which cell fate is independent of determinant segregation. In the *Drosophila* testes, positional placement of the GSCs within the stem cell niche determines fate. There, proximity of GSCs next to the hub cell maintains stem cell identity [70,71]. Adherens junctions provide a positional cue to orient spindles away from the hub such that gonialblasts are positioned out of the niche and adopt a differentiating fate [43,72,73]. In some cases, such as in zebrafish retina, even cell divisions can be dispensable. Rather, the proximity of progenitor neuron nuclei to either the apical or basal surface during interkinetic nuclear migration balances proliferative or neurogenic fates, respectively, in response to a Notch gradient [74]. Additionally, as evidenced in the chick neuroepithelium, cell fate choices can involve intrinsic features of the cells themselves, dependent upon neither spindle orientation nor cellular positioning [75].

With this comparative outlook, the ACDs that occur in the embryonic epidermis are poised to rely at least in part on the microenvironment to produce the desired differential outcome in cell fate. It seems unambiguous that Notch plays a considerable role in epidermal development, where it promotes differentiation [76–79]. While Notch is coupled to ACDs early in embryonic skin development, it is possible that the mature epidermis establishes a Notch signalling transitional zone, creating a differing response by the basal and suprabasal layers [4]. Notch signalling is transduced when a signal-presenting cell that expresses the ligand interacts with the Notch receptor on the surface of the signal-receiving cell. When ligand and receptor interact, the Notch intracellular domain (NICD) of the receptor is cleaved, and translocates to the nucleus where it functions as a transcriptional co-activator.

Interestingly, in the mature epidermis, Notch ligands Delta-like1 and Jagged2 are expressed in the basal layer, while the suprabasal layer is enriched for Jagged1, receptors Notch1, Notch2 and Notch3, and the Notch target, Hes1 [80]. In this regard, microenvironmental cues are positioned to dominate later in development, lessening the need for ACDs in adult skin, while ACDs may need to be more important during embryogenesis, where there is differential expression of Notch ligands basally and suprabasally. As attractive as this hypothesis might seem, the intricate coupling of intrinsic and extrinsic processes during embryogenesis makes dissecting their roles difficult. For instance, even in embryonic tissue where ACDs are prevalent, depletion of LGN results in a concomitant reduction of spinous layers and their expression of Notch 3 and Hes1, and Notch signalling [4]. That said, Notch appears to be the predominant downstream effector of LGN-dependent asymmetric divisions in the epidermis, because reintroduction of NICD in cells depleted of LGN can rescue epidermal stratification [4].

## 8. Conclusions and outstanding questions

Significant headway has been made in understanding the processes that drive the morphogenesis of the epidermis. Progenitor divisions in the basal layer of the epidermis drive not only its lateral expansion but contribute to its stratification as well. The perpendicular orientation of the mitotic spindle in asymmetrically dividing basal cells positions progeny within different cell strata, priming cell fate acquisition through Notch-dependent differentiation.

Yet, just as our understanding expands, so do our questions. What are the cues that prompt a symmetric and lateral division versus an asymmetric and perpendicular one? If cortical crescents of mInsc and LGN appear partway into the stratification timeline, are there additional mechanisms that guide spindle orientation during the earliest stages of stratification? What are the mechanisms regulating the expression of ACD machinery in the epidermis? And do oriented divisions also contribute to the complex architecture of the developing hair follicles?

Additionally, what is the consequence of altering the balance between symmetric and asymmetric divisions? How are cell fate decisions altered on an individual cell basis, when angled divisions are not strictly parallel or perpendicular? The answers to these and many other questions await future investigations aimed at heightening our understanding of how stem cells divide and dissecting the cues that guide them.
